# Prenatal high-dose vitamin D_3_ supplementation has balanced effects on cord blood Th1 and Th2 responses

**DOI:** 10.1186/s12937-016-0194-5

**Published:** 2016-08-09

**Authors:** Evana Akhtar, Akhirunnesa Mily, Ahsanul Haq, Abdullah Al-Mahmud, Shams El-Arifeen, Abdullah Hel Baqui, Daniel E. Roth, Rubhana Raqib

**Affiliations:** 1 Immunobiology, Nutrition and Toxicology Laboratory, Infectious Diseases Division, icddr,b, Dhaka, 1212 Bangladesh; 2 Maternal and Child Health Division, icddr,b, Dhaka, 1212 Bangladesh; 3 International Center for Maternal and Newborn Health, Department of International Health, The Johns Hopkins Bloomberg School of Public Health, 615 North Wolfe Street, Baltimore, MD 21205 USA; 4 Department of Pediatrics, Hospital for Sick Children and University of Toronto, 555 University Avenue, Toronto, ON M5G 1X8 Canada

**Keywords:** Vitamin D_3_, Cytokines, T lymphocytes, Neonates, Pregnant women, Cord blood

## Abstract

**Background:**

Antenatal vitamin D_3_ (vitD_3_) supplementation significantly increases maternal and neonatal 25-hydroxyvitamin D_3_ (25(OH)D_3_) concentration, yet the effect of an improvement in maternal-fetal vitamin D status on the neonatal immune response is unclear.

**Method:**

To assess the effect of prenatal vitD_3_ supplementation on cord blood T cell function, healthy pregnant Bangladeshi women (*n* = 160) were randomized to receive either oral 35,000 IU/week vitD_3_ or placebo from 26 to 29 weeks of gestation to delivery. In a subset of participants (*n* = 80), cord blood mononuclear cells (CBMC) were cultured, non-adherent lymphocytes were isolated to assess T cell cytokine responses to phytohemagglutinin (PHA) and anti-CD3/anti-CD28 (iCD3/iCD28), measured by multiplex assay. In 12 participants, lymphocyte gene expression profiles were analyzed by PCR array.

**Result:**

In supplemented group, increased concentrations of IL-10 (*P* < 0.000) and TNF-α (*P* = 0.05) with iCD3/iCD28 stimulation and IFN-γ (*p* = 0.05) with PHA stimulation were obtained compared to placebo group. No differences in the gene expression profile were noted between the two groups. However, PHA stimulation significantly induced the expression of genes encoding Th1 and Th2 cytokines and down-regulated a number of genes involved in T-cell development, proliferation and differentiation of B cells, signal transduction pathway, transcriptional regulation and pattern recognition receptors (PRRs) in the vitamin D group (vitD group).

**Conclusion:**

Third-trimester high-dose vitD_3_ supplementation in healthy pregnant women had balanced effects on biomarkers of cord blood Th1 and Th2 responses.

**Trial registration:**

ClinicalTrials.gov (NCT01126528).

**Electronic supplementary material:**

The online version of this article (doi:10.1186/s12937-016-0194-5) contains supplementary material, which is available to authorized users.

## Introduction

The importance of adequate vitamin D status during pregnancy has been suggested by observational studies demonstrating associations between low 25(OH)D_3_ and adverse birth outcomes, childhood infections, and atopy [1, 2]. Several studies have demonstrated that prenatal vitD_3_ supplementation or vitD_3_ intake during pregnancy was associated with reduced risk of wheezing and asthma in young [1, 3–5]. In contrast, no association [6, 7] or negative effects of high vitamin D intake in pregnancy on the risk of eczema, asthma and wheezing in the offspring have also been shown [8]. There is no consensus on the cutoff value for vitamin D deficiency and the optimum dosage for supplementation during pregnancy remains controversial.

Vitamin D is hypothesized to be an important regulator of immune and inflammatory responses [9]. Maternal vitamin D status during pregnancy may affect the fetal immune system and contribute to the risk of development of immune-mediated diseases and infection in the offspring. Normal pregnancy is typically associated with a predominance of Th2- and Th3-type cytokine profile and a relative suppression of T helper type 1 (Th1) response in the mothers [10, 11]. Various in vitro studies of the effects of vitamin D on the T-cell phenotype have shown that active vitamin D_3_ (1,25(OH)_2_D_3_) suppresses production of Th1 cytokines and IL-17 [12, 13] and promotes Th2 responses [14, 15] and T regulatory cells (Treg) to maintain a balance and prevent exacerbated immune responses [16, 17]. However, it is unknown whether vitD_3_ induced Th1 suppression and Th2 promotion occur in vivo, particularly in the context of fetal immune ontogeny.

We aimed to evaluate the effect of prenatal vitD_3_ supplementation on T lymphocyte activation pathways and cytokine responses in cord blood. Earlier we have shown in a double-blind, placebo-controlled trial, supplementation with 35,000 IU/week of vitD_3_ during the 3^rd^ trimester of pregnancy among Bangladeshi women significantly increases the mean 25(OH)D_3_ concentration in cord blood compared to the placebo group (103 versus 39 nmol/L) [18]. In a sub-set in the same trial cohort, we studied the impact of vitamin D status on the expression profile of cytokines by stimulated T lymphocytes and on T cell activation pathway as a key component of adaptive immunity.

## Methods

### Study design and participants

The Antenatal Vitamin D in Dhaka (AViDD) study was a randomized, double-blinded, placebo-controlled trial to evaluate the effect of high-dose of vitD_3_ (cholecalciferol) supplementation during 3rd trimester of pregnancy on maternal and cord serum (25(OH)D_3_) concentration and primary biochemical efficacy outcomes [18]. Briefly, pregnant women were enrolled at the Shimantik Urban Primary Health Care Project Maternity Centre, a non-governmental facility that provides basic antenatal and obstetric services in a low-income community. Data collected from each participant included maternal education, occupation, construction features of the dwellings, gestational age at delivery, delivery mode, birth weight and sex of babies. Inclusion criteria were pregnant women with gestational age of 26 to 29 weeks, age range 18 to <35 years, currently residing in Dhaka, plans to stay in Dhaka throughout pregnancy up to one month post-delivery, and plans to deliver at the maternity centre. Study participants were allocated to receive a weekly dose of either 35,000 IU of vitD_3_ (Vigantol Oil, Merck KGaA, Germany; vitD group) or placebo oil (Miglyol oil, Merck; placebo group) until delivery. The study was approved by two committees of icddr,b, the Research Review Committee (RRC) and the Ethical Review Committee (ERC) (Protocol# PR-09058). The Johns Hopkins Bloomberg School of Public Health (Baltimore, USA), and the Hospital for Sick Children (Toronto, Canada). Written informed consent was obtained from all eligible participants.

A total of 160 pregnant women were enrolled in the original trial. The subset of women (*n* = 80; 40 from placebo group and 40 from vitD group) selected for the current study were those for whom adequate CBMCs were available for stimulation assays for cytokine analysis. For analysis of lymphocyte activation pathways by PCR array method, we selected 6 participants from the vitD group and 6 from the placebo group based on availability of complete data and adequate CBMC counts.

### Vitamin D status assessment

Serum 25(OH)D_3_ was measured by high-performance liquid chromatography tandem mass spectroscopy (LC-MS/MS), which showed that only 25(OH)D_3_ was obtained in the serum samples, 25(OH)D_2_ was not detectable in any serum samples [18, 19]. Details of the measurement procedure have been described elsewhere [18, 19]. There is no standard classification of vitamin D status based on 25(OH)D_3_ concentrations in cord blood; thus, for the purpose of the present study we stratified the participants into four groups based on serum 25(OH)D concentrations as done earlier [20] :(1) ≥76 nmol/l (high); (2) 50–75 nmol/l (moderate); (3) 30–49 nmol/l (low); (4) <30 nmol/l (very low).

### Cord blood collection, plasma and non-adherent lymphocyte isolation

Venous cord blood was collected immediately after delivery and transferred to the icddr,b laboratory in Dhaka for same-day processing (within 2–18 h). Cord blood plasma and CBMCs were separated from whole blood by Ficoll-Paque (Amershan-Pharamcia Biotech, Sweden) density gradient centrifugation. The isolated CBMCs were re-suspended in RPMI 1640 medium (Gibco, Invitrogen, Grand Island, NY, USA) containing 10 % autologous plasma and cultured in tissue culture plates (NUNC, Roskilde, Denmark) for 2 h to separate non-adherent cells from adherent cells that stick to the plastic surface of the tissue culture plates. The non-adherent lymphocytes, which consisted predominantly of 70–75 % of T lymphocyte (CD3), 20–23 % of B lymphocytes (CD19) and 5–7 % of Natural killer cells (CD16) were separated from adherent monocytes (CD14) (10–15 % in total CBMC), and cultured in presence of stimulants (T cell mitogen Phytohemagglutinin (PHA) or agonistic antibodies to T-cell receptor).

### Stimulation of lymphocytes

Stimulation with anti-CD3 and anti-CD28 antibodies predominantly activates T lymphocytes. PHA activates T cells by binding to cell membrane glycoproteins, including the T cell receptor (TCR) CD3 complex. One fraction of lymphocytes was stimulated with anti-human CD3e (purified mouse Monoclonal IgG1, Clone UCHT1; R&D Systems, Minneapolis, MN 55413, USA), plus anti-human CD28 (purified mouse Monoclonal IgG1, Clone 37407) (iCD3/iCD28) 5 μg/mL each), the 2nd fraction of lymphocytes was stimulated with PHA (Sigma (St Louis, MO, USA; 5 μg/mL), and the 3rd fraction of lymphocytes was cultured as control (without any stimulation) in 96-well tissue culture plates (NUNC, Roskilde, Denmark) for 48 h at 37 °C in 5 % CO2 incubator. After incubation, all of the culture supernatant from each fraction of lymphocytes was collected and used for assessment of cytokine secretion by Cytometric Bead Array (CBA). After PHA stimulation the stimulated lymphocytes were harvested by centrifugation, stored in RNAlater (QIAGEN GmbH, Hilden, Germany) for further use for evaluating T lymphocyte activation markers by PCR array.

### Assessment of cytokines in lymphocyte supernatant

The Human Th1/Th2/Th17 Cytometric Bead Array (CBA) Kit (BD Biosciences, San Jose, CA) was used for measurement of cytokines in culture supernatant from iCD3/iCD28- and PHA-stimulated lymphocytes. Cytokines IL-2, IL-4, IL-6, IL-10, TNF-α, IFN-γ and IL-17A were quantified. BD FACS Caliber II was used for acquisition and CBA FCAP Array (Version 1.0.1) software was used for result analysis. The lower limit of detection (LOD) was 2.6, 4.9, 2.4, 4.5, 3.8, 3.7 and 18.9 pg/mL for IL-2, IL-4, IL-6, IL-10, TNF-α, IFN-γ and IL-17A respectively. Data were expressed as ratio of cytokines in the stimulated lymphocytes by unstimulated control lymphocytes. T helper 1 (Th1) cytokines include IL-2, IFN-γ and TNF-α and T helper 2 (Th2) cytokines include IL-4, IL-6 and IL-10. IL-17A is secreted by Th17 cells.

### T and B cell activation pathway

Extractions of mRNA from both PHA-stimulated and unstimulated lymphocytes were performed using RNeasy Mini kit according to the manufacturer’s instructions (Qiagen GmbH, Hilden, Germany). Using the RT2 First Strand Kit (SA Bioscience, Life Technologies, Carlsbad, California), cDNA was prepared from mRNA in a CFX96TM real time system (C1000TM Thermal cycler, Bio-Rad Life Science Research, Hercules, CA). At least 1.0 μg RNA was added to ensure a maximum number of positive calls in the PCR Array System. The cDNA was mixed with an appropriate RT2 SYBR Green master mix and the mixture was added to the wells of RT2 Profiler PCR Array (SA Bioscience). PCR was performed according to the following protocol: one cycle of 10 min at 95 °C followed by 40 cycles of 15 s at 95 °C, and 1 min at 60 °C. Values were exported to a template excel file provided by SABiosciences for data analysis. The C_T_ values of genes were normalized by the average C_T_ values of five housekeeping genes [Beta-2-microglobulin (B2M), Hypoxanthine phosphoribosyltransferase 1 (HPRT1), Ribosomal protein L13a (RPL13A), Glyceraldehyde-3-phosphate dehydrogenase (GAPDH), Actin, beta (ACTB)]. The fold change (2^(-Delta Delta Ct (∆∆CT)) is the normalized gene expression in test sample (stimulated cells) divided by the normalized gene expression in the control sample (unstimulated cells). Fold regulation represents fold change results in a biologically meaningful way, with a positive fold indicating up-regulation and negative fold indicating down-regulation of the gene. Gene expression data (∆CT) within acceptable range (25–30 threshold cycle values) were included in the analysis.

PCR array was performed for the assessment of 84 predefined genes of T- and B cell activation pathway that included genes involved in T and B cell activation, proliferation and differentiation as well as genes regulating Th1 and Th2 development and T cell polarization. Moreover, genes involved in the activation of macrophages, neutrophils, and natural killer cells were also included in this system. For each participant, gene expression data was obtained for unstimulated and PHA-stimulated lymphocytes within a group; direct comparison between vitD and placebo groups was not performed in the software provided by SABiosciences.

### Statistical methods

Statistical analyses were performed using IBM SPSS Statistics for Windows (version 20; Armonk, NY: IBM SPSS corp.; 2011) and Stata/IC, version 13 (StataCorp, Texas, USA). P values <0.05 were considered statistically significant. Independent sample t-test was used to estimate the mean difference of baseline characteristics. The primary outcome measure for each participant was the ratio of cytokine concentration in PHA- or iCD3/iCD28-stimulated to unstimulated cell cultures. Data were described by their ranges, mean and standard deviation. Cytokine concentrations did not follow normal distributions therefore cytokine data were normalized by log transformation. Henceforth, cytokine concentration will represent normalized cytokine ratio. Linear regression model was used to evaluate the influence of vitD_3_ supplementation on cytokine concentrations in vitD group compared to placebo. The model was adjusted for covariates that were associated with biologically relevant outcomes or those that changed the effect estimate by more than 5 %. The potential covariates were maternal age, occupation, education, gestational age, delivery mode, birth weight of infants, child sex and baseline serum 25(OH)D_3_ level. PCR array data were analyzed in the software provided by SABiosciences (www.SABiosciences. com/pcrarraydataanalysis.php) comparing stimulated versus unstimulated cells within a group. The p-values were calculated based on Student’s t-test of the replicate of 2^(-Delta CT) values for each gene in the unstimulated and stimulated subjects within each supplemental group (*n* = 6).

## Results

### Participant characteristics

In the current study, the mean age of women, maternal occupation, education, construction features of living abode, gestational age, delivery mode and serum 25(OH)D_3_ concentration at baseline were similar among the pregnant women in the vitD and placebo groups (Table 1). The newborns in the vitD group were not significantly different from those in the placebo group in birth weight and male female ratio. The baseline demographic features of the participants in the original study cohort (*n* = 160) were similar to those in the present cohort (*n* = 80) as well as to the PCR-Array sub-group (*n* = 12) (Additional file 1: Table S1). Information for delivery mode was available for 147 out of 160 participants in the original study (92 %) [18] (Additional file 1: Table S1). Ceasarean delivery was high in participants of both the original cohort (60 %, 88 of 147) and the present cohort (69 %, 55 of 80) and in <50 % cases the rationale for performing caesarean deliveries was due to fetal distress including reduced fetal movement and breech/transverse fetal presentation (Additional file 1: Table S2). In the PCR array group, 11 participants were delivered by C-section while only one was delivered vaginally (Table 1).

**Table 1:** Demography of the participants supplemented with vitamin D_3_ or placebo

	Total group			Sub-group of PCR array		
Variables	vitD group (*n* = 40)	Placebo group (*n* = 40)	*p*	vitD group (*n* = 6)	Placebo group (*n* = 6)	*p*
	Mean ± SD	Mean ± SD		Mean ± SD	Mean ± SD	
^a^Maternal age, years	22.43 ± 3.75	22.85 ± 1.47	0.6	21.67 ± 3.93	20.17 ± 1.47	0.4
^a^Gestational age at birth, week	39.16 ± 2.38	38.39 ± 2.61	0.6	39.97 ± 2.44	40.18 ± 2.24	0.9
^b^Delivery mode n (%)						
Vaginal	13 (30 %)	12 (32.5 %)	0.8	0 (0.0 %)	1 (16.7 %)	0.2
C-section	27 (70 %)	28 (67.5 %)		6 (100.0 %)	5 (83.3 %)	
^a^Birth weight, gm	2865.25 ± 522.46	2865.50 ± 357.83	0.9	2916.67 ± 636.92	2843.33 ± 308.65	0.8
^c^Male:female	16:24	18:22	0.4	3:3	2:4	1.0

Data expressed as means ± standard deviations and or number with percentage in parentheses.

^a^Student’s t test was used for calculating p values for maternal age, gestational age at birth and birth weight

^b^P values were calculated for delivery mode between two supplementation using χ^2^test

^c^Fisher Exact test was applied for comparisons between the two group

Cord serum 25(OH)D_3_ concentrations were significantly higher in the vitD group compared to the placebo group, as expected based on the full trial results [18]. When neonates were stratified into 4 categories based on serum 25(OH)D_3_ concentrations, 88 % participants in the vitD group were in the high category as opposed to 8 % in the placebo group (Table 2). Among the participants included in the PCR-array sub-groups, all 6 neonates in the vitD group were in the moderate to high category (85.33 ± 16.0 nmol/L), while all placebo neonates belonged to the low category (38.83 ± 8.4 nmol/L) (data not shown).

**Table 2:** Concentration of 25(OH)D_3_ in baseline and cord blood samples

25(OH)D_3_ nmol/L	Placebo (*n* = 40)	vitD (*n* = 40)	*p*-value^a^
Baseline	45.6 ± 21.4	42.6 ± 17.2	0.495
Cord blood	36.8 ± 16.2	101.2 ± 29.9	<0.000
Stratification of 25(OH)D_3_			
Baseline			
< 30	11 (27.5 %)	12 (30.0 %)	
30–49	14 (35.0 %)	18 (45.0 %)	
50–75	10 (25.0 %)	7 (17.5 %)	
≥ 76	5 (12.5 %)	3 (7.5 %)	
Cord blood			
< 30	17 (42.5 %)	1 (2.5 %)	
30–49	17 (42.5 %)	1 (2.5 %)	
50–75	3 (7.5 %)	3 (7.5 %)	
≥ 76	3 (7.5 %)	35 (87.5 %)	

Data expressed as means ± standard deviations and or numbers with percentages in parentheses

^a^Independent sample t test was used to assess the comparison of serum 25(OH)D_3_ concentrations between the two groups

### Cytokine concentrations in stimulated cord blood lymphocytes

In the stimulation experiments, concentrations of all cytokines except for IL-4 were above the LOD. More than 90 % of the participants from each treatment group exhibited less than LOD for IL-4 concentration; thus, data for IL-4 was not included in further analysis. Linear regression analysis demonstrated higher concentrations of IL-10 and TNF-α in the vitD group after iCD3/iCD28 stimulation compared to the placebo (Table 3). Similarly, higher concentrations of IFN-γ were obtained in the vitD group after PHA-stimulation compared to the placebo group. No significant differences were observed for the other cytokines.

**Table 3:** Regression analysis of cytokines in vitD group compared to placebo group

	Unadjusted		Adjusted	
Cytokine	β (95 % CI)	*p*-*value*	β (95 % CI)	*p*-*value*
Anti-iCD3/iCD28				
IL-2	−0.10 (−0.77, 0.56)	0.76	−0.26 (−0.93, 0.42)	0.44
IL-6	0.17 (−0.13, 0.47)	0.27	0.23 (−0.09, 0.54)	0.16
IL-10	0.33 (0.02, 0.65)	0.03	0.62 (0.44, 0.80)	<0.000
TNF-α	0.41 (0.002, 0.81)	0.04	0.38 (−0.00, 0.84)	0.05
IFN-γ	0.40 (−0.14, 0.93)	0.14	0.42 (−0.17, 0.95)	0.17
IL-17A	0.23 (−0.06, 0.52)	0.12	0.24 (−0.08, 0.56)	0.15
PHA				
IL-2	−0.42 (−1.13, 0.29)	0.24	−0.30 (−0.96, 0.36)	0.36
IL-6	0.17 (−0.13, 0.47)	0.27	0.19 (−0.11, 0.48)	0.21
IL-10	0.16 (−0.27, 0.58)	0.46	0.21 (−0.19, 0.62)	0.29
TNF-α	0.20 (−0.25, 0.65)	0.38	0.25 (−0.18, 0.67)	0.25
IFN-γ	0.53 (−0.09, 1.15)	0.09	0.59 (−0.006, 1.17)	0.05
IL-17A	0.15 (−0.51, 0.81)	0.65	0.21 (−0.45, 0.87)	0.53

Data were given as beta (β) and 95 % confidence interval; β, regression coefficients. Adjusted for Delivery mode, gestational week and baseline 25(OH)D_3_ level

When Th1-to-Th2 ratios were assessed using IL-10 or IL-6 as the denominator (e.g. IL-2/IL-10, IFN-γ/IL-10, TNF-α/IL-10), no significant associations were obtained between vitD and placebo groups (data not shown).

### Prenatal vitamin D supplementation and T and B cell activation pathways

**Fig. 1:**
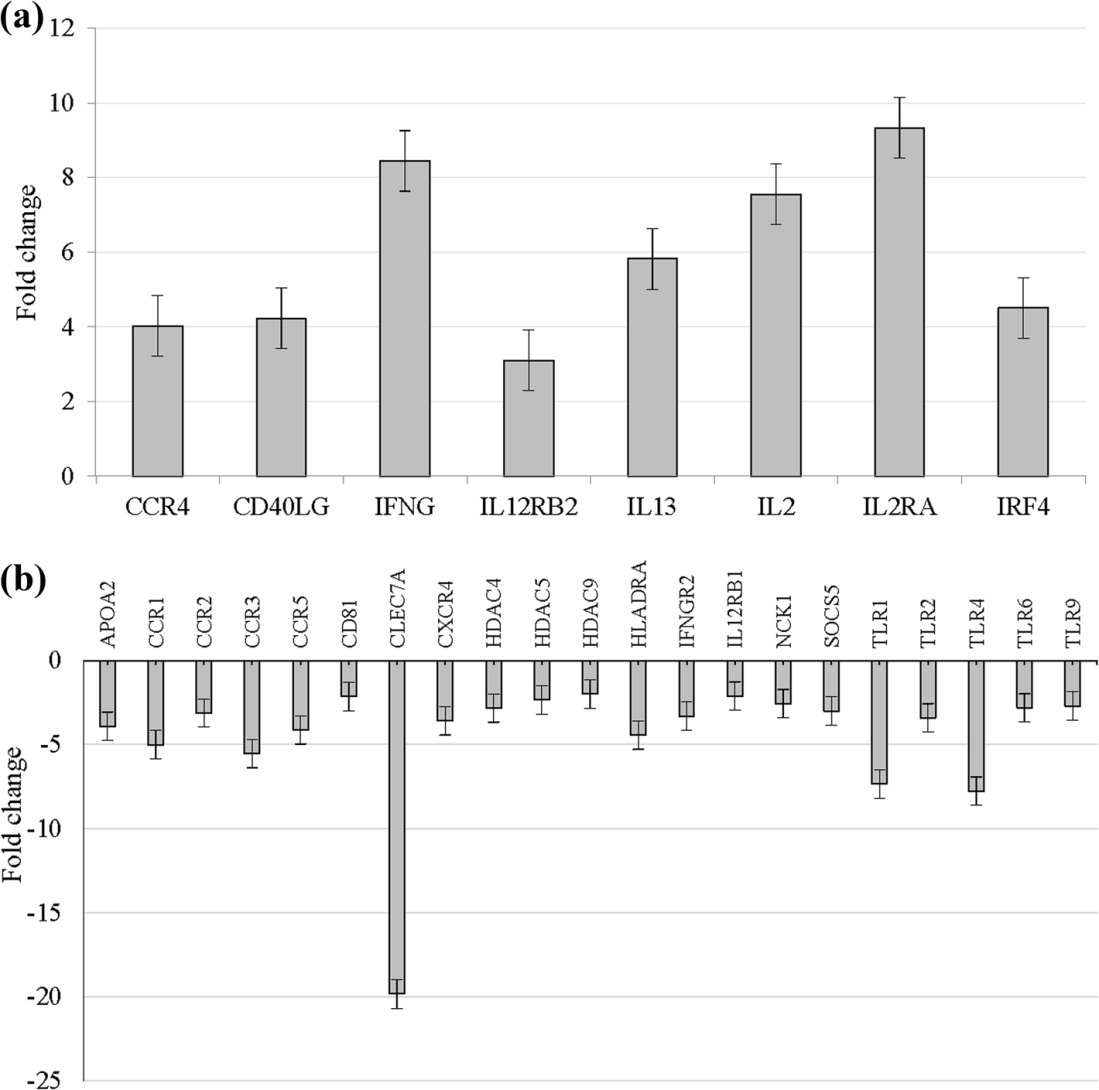
Fold change of gene expression level after stimulation with phytohemagglutinin (PHA) in the vitD group. Genes increased or decreased in the stimulated cells compared to the unstimulated cells by ≥2 were showed in the figure. Among 84 genes of T and B cell pathway. **a** only 8 genes were upregulated, while (**b**) 21 genes were down regulated. Results were from 6 participants

Within each intervention group, comparisons were made between genes from unstimulated and stimulated lymphocytes. In the vitD group, PHA stimulation of lymphocytes significantly induced expression of genes encoding Th1 cytokines IFN-γ, IL-2, IL-2 receptor (IL-2R), Th2 cytokine IL-13 and IL-12RB2 [receptor for IL-12, that is up-regulated by IFN-γ] compared to unstimulated cells. There was also significant induction of CD2 [an adhesion molecule expressed on T & NK cells that also acts as a co-stimulatory molecule], CD40LG[primarily expressed on activated T cells; regulates B cell function, mediates B-cell proliferation and immunoglobulin class switching], IRF4 [a transcription factor essential for the development of Th2 cells, IL-17 producing cells and IL-9 producing Th9 cell (associated with Th2 immunity)], CCR4 (Chemokine (C-C motif) receptor 4 [preferentially expressed by FOXP3+ Treg (Th2) cells] (Fig. 1a). Compared to unstimulated control lymphocytes, PHA stimulation significantly down-regulated many C-C chemokine receptors (CCR1, CCR2, CCR3 and CCR5), multiple Toll-like receptors (TLRs)(TLR1, TLR2, TLR4, TLR6, TLR9), CXCR4, CLEC7A (C-type lectin domain family 7, member A), SOCS5 (Suppressor of cytokine signaling 55), ICOSLG (Inducible T-cell co-stimulator ligand), CD3D and CD3G (subunits in CD3-T Cell Receptor complex), CD8B (B subunit on cytotoxic T cells), CD81 (expressed on T lymphocytes), TGF-β, IFNGR1, IFNGR2 (receptors of IFN-γ), HLA-DR, NCK1, NCK2, and histone deacetylases (HDAC4-9) (Fig. 1b) (Table 4).

**Table 4:** Description of the functions of some of the genes in the vitD group as analyzed by PCR array method

Gene symbol	Description	Fold up- or down -regulation	*P*-*value*	Function
APOA2	Apolipoprotein A-II	−3.93	0.001	Apolipoproteins function as structural components of lipoprotein particles
CCR1	Chemokine (C-C motif) receptor 1	−5.01	0.055	Expressed on peripheral blood lymphocytes, specifically memory T-cells; binds to multiple inflammatory chemokines
CCR2	Chemokine (C-C motif) receptor 2	−3.13	0.001	Expressed on activated memory T cells & B cells
CCR3	Chemokine (C-C motif) receptor 3	−5.54	0.007	Receptor for multiple inflammatory/inducible CC chemokines;
				Expressed on Th1 and Th2 cells, CCR3 plays a role in allergic reactions
CCR4	Chemokine (C-C motif) receptor 4	4.03	0.001	Expressed on Th2 cells, preferentially by CD45RA^+^ naïve FOXP3^+^ Treg cells
CCR5	Chemokine (C-C motif) receptor 5	−4.14	0.029	Expressed on activated/memory Th1 lymphocytes.
CD2	CD2 molecule	1.47	0.042	An adhesion molecule expressed on T & NK cells that acts as a co-stimulatory molecule
CD3D	CD3d molecule, delta (CD3-TCR complex)	−1.62	0.023	CD3 subunits involved in T cell activation/signaling through TCR
CD3G	CD3g molecule, gamma (CD3-TCR complex)	−1.76	0.031	CD3 subunits involved in T cell activation/signaling through TCR
CD40LG	CD40 ligand	4.23	0.008	Plays important role in T cell dependent immune response
				Primarily expressed on activated T cells and regulates B cell function, mediates B-cell proliferation and immunoglobulin class switching
CD81	CD81 molecule	−2.15	0.026	mediate signal transduction events; on T cells CD81 associates with CD4 and CD8 and provides a co-stimulatory signal with CD3;
				CD81 expression by T cells enhances cognate T–B cell interactions as well as intracellular activation pathways leading to Th2 polarization.
CD8B	CD8b molecule	−1.82	0.016	B subunit on cytotoxic T cells for cellular interaction
CLEC7A	C-type lectin domain family 7, member A	−19.84	0.020	Expressed on dendritic cells, monocytes, macrophages and B cells; plays a role in innate immunity through pathogen recognition receptor (PRR) can operate as a co-stimulatory molecule via recognition of an endogenous ligand on T-cells, which leads to cellular activation and proliferation. CLEC7A can bind both CD4+ and CD8+ T cells
CXCR4	Chemokine (C-X-C motif) receptor 4	−3.59	0.002	acts in lymphocytes chemotaxis; a chemokine receptor;
				Expressed on T and B cells; central role of CXCR4 in confining migrating B cells to the proper target sites or an important role for CXCR4 in regulating homeostasis of B cell compartmentalization and humoral immunity.
HDAC4	Histone deacetylase 4	−2.84	0.029	HDAC4 regulates bone and muscle development & promotes healthy vision.
				Specific and critical functions in transcriptional regulation & cell cycle progression; have histone and nonhistone protein substrates
HDAC5	Histone deacetylase 5	−2.35	0.033	transcriptional regulation
HDAC9	Histone deacetylase 9	−2.00	0.044	reduces transcriptional regulation after stimulation
HLADRA	Major histocompatibility complex, class II, DR alpha	−4.44	0.009	Expressed on B cells & helper T cells (activated CD4^+^ CD25^+^ Treg cells)
ICOSLG	Inducible T-cell co-stimulator ligand	−1.64	0.031	Co-stimulatory signal for T-cell & B-cell proliferation; ICOS is selectively expressed on Th2 cells.
				ICOS, which is selectively expressed on Th2‐polarized T cells, predominantly enhance Th2 cytokine production, indicating that co‐stimulatory molecules influence the polarization process to Th1 or Th2 phenotypes.
IFNG	Interferon, gamma	8.45	0.024	Th1 cytokine; critical for innate and adaptive immunity against viral and intracellular bacterial infections and for tumor control; important activator of macrophages.
IFNGR1	Interferon gamma receptor 1	−1.92	0.050	Activity of IFN-γ is reduced due to low availability of IFNgR
IFNGR2	Interferon gamma receptor 2 (interferon gamma transducer 1)	−3.31	0.001	Activity of IFN-γ is reduced due to low availability of IFNgR
IGBP1	Immunoglobulin (CD79A) binding protein 1	−1.45	0.012	B cells proliferation and differentiation
IL12RB1	Interleukin 12 receptor, beta 1	−2.12	0.024	Activities of natural killer cells and cytotoxic T lymphocytes; a link between IL-2 and the signal transduction of IL-12 in NK cells.
				(The protein encoded by this gene is a type I transmembrane protein, which acts in signal transduction)
IL12RB2	IL-12 receptor, beta 2	3.10	0.002	Contributes to the inflammatory response and host defense
IL13	Interleukin 13	5.82	0.040	expressed by Th2 cells, Has anti-inflammatory properties, acts in Th2 responses
IL2	Interleukin 2	7.55	0.002	Increase proliferation, differentiation of effector T cells and T-reg cells
IL2RA	Interleukin 2 receptor, alpha	9.34	0.000	an important modulator of immunity
IRF4	Interferon regulatory factor 4	4.51	0.000	a transcription factor essential for development of Th2 cells, IL-17 producing cells & IL-9 producing Th9 cell associated with Th2 immunity
KLF6	Kruppel-like factor 6	−1.97	0.000	function in mitosis, meiosis and transport of cellular cargo
NCK1	NCK adaptor protein 1	−2.57	0.002	associated with bone metabolism, involved with actin cytoskeletal remodeling, signal transduction
NCK2	NCK adaptor protein 2	−1.93	0.015	Nck1 and Nck2 are two highly related adaptor proteins downstream of the TCR
SOCS5	Suppressor of cytokine signaling 5	−3.01	0.001	The SOCS proteins negatively regulate cytokine and Toll-like receptor- (TLR-) induced signaling in the inflammatory cells.
				TLR signals are regulated by molecules such as TOLLIP and SOCS5
TGFB1	Transforming growth factor, beta 1	−1.62	0.043	Expressed by macrophages and CD4+ T.
				TGF-Beta is a potent immunosuppressor, all immune cell lineages secrete TGF-Beta, it controls the proliferation, differentiation of these cells. Perturbation of TGF-Beta signaling is linked to autoimmunity, inflammation and cancer
TLR1	Toll-like receptor 1	−7.36	0.000	Expressed on of macrophages, neutrophils, B lymphocytes
				They recognize pathogen-associated molecular patterns (PAMPs) that are expressed on infectious agents, and mediate the production of cytokines necessary for the development of effective immunity.
TLR2	Toll-like receptor 2	−3.42	0.068	Expressed on macrophages, neutrophils, B lymphocytes, dendritic cells
TLR4	Toll-like receptor 4	−7.78	0.055	B lymphocytes; monocytes/macrophages, neutrophils, dendritic cells, Mast cells, Intestinal epithelium
TLR6	Toll-like receptor 6	−2.82	0.004	Activated Treg cells, B lymphocytes, monocytes/macrophages, Mast cells
TLR9	Toll-like receptor 9	−2.71	0.079	Monocytes/macrophages, Plasmacytoid dendritic cells, B lymphocytes

Data provided only for those genes that had expression (∆CT) within acceptable range (25-30 threshold cycle values)

In the placebo group, no significant changes were noted in the gene expression in PHA stimulated lymphocytes compared to un-stimulated cells (Additional file 1: Table S3).

## Discussion

We previously showed that supplementation with vitD_3_ during the 3rd trimester of pregnancy improved maternal and cord blood vitamin D status [18] and reduction of antibacterial peptide LL-37 in monocytes [20]. Here, we report that the antenatal vitD_3_ supplementation resulted in induction of both Th1 and Th2 cytokines after stimulation of cord blood lymphocytes. In the vitD group, the gene expression profile in stimulated lymphocytes demonstrated down-regulation of genes involved in transcriptional regulation, and components of the innate immune response that are involved in recognizing and defending against invading pathogen.

There is a scarcity of in vivo data in humans during pregnancy showing effects of vitamin D supplementation on neonatal immune function. In vitro studies have shown that active vitamin D_3_ (1,25(OH)_2_D_3_) preferentially inhibits expression of Th1 cytokines while simultaneously inducing Th2 cytokines [9, 14, 15]. Again, other studies have shown contrasting role or no preferential effect for vitD_3_. For example, in cultured human trophoblasts, 1,25(OH)_2_D_3_ down-regulated IL-10 expression under normal and experimental inflammatory conditions and directly inhibited TNF-α and IL-1β stimulation of IL-10 [21]. Supplementation of healthy women with oral vitD_3_ for 6 months did not lead to significant alterations in expression of IFN-γ and IL-4 cytokines compared to placebo group, nor were there any effects on transcription factors T-bet and GATA3, that regulate the Th1/Th2 fate of CD4^+^T cells [22]. The present study demonstrated in vivo effects of prenatal vitD_3_ supplementation on induction of not only Th2 cytokines but also Th1 cytokines, without a clear predominance of either T cell phenotype. Similarly, in the vitD group PCR array analysis demonstrated increased transcript levels of Th1 and Th2 cytokines as well as transcription factor IRF4 that regulates development of Th2, IL-17 and IL-9 producing cells.

Active vitamin D can inhibit in vitro proliferation and differentiation of B cells into plasma cells as well as antibody production [23, 24]. Increased frequency of B cells and antibody production has been reported in vitamin D deficient women and their neonates (cord blood) compared to the vitamin D sufficient group [16]. In the present study, ICOSLG and IGBP1 genes that promote B-cell proliferation and differentiation into plasma cells were down-regulated, while, CD40LG that mediates B-cell proliferation and immunoglobulin class switching was up-regulated in the vitD group.

The findings of several responses to T cell stimulation that were robust in the vitD group but non-significant in the placebo group were consistent with previous literature. For example, CCR4 is preferentially expressed by naïve Foxp3^+^ Treg cells which require high levels of IL-2 for expansion in vitro [25]. Our findings of increased expression of CCR4 and IL-2 in vitD group was in accordance with data from in vivo studies showing positive association between Foxp3^+^ Treg cells (number and frequency) and serum 25(OH)D_3_ [16, 26–28] but not with active vitD_3_ [27]. TGF-β plays an important role in generation of Foxp3^+^Tregs from naive CD4^+^T cells albeit in presence of low concentration of 1,25(OH)_2_D_3_ [25]. We found decreased levels of TGF-β transcripts in stimulated lymphocytes in the vitD group.

Klug-Micu et al showed that two parallel T-cell-mediated mechanisms, IFN-γ released by T cells and induction of CD40L on T cells, trigger antimicrobial responses against intracellular pathogens through a common vitamin D-dependent antimicrobial pathway [29]. We also found up-regulation of CD40L as well as IFN-γ expression in activated lymphocytes in the vitD group.

Vitamin-D-mediated immunity provides feedback control that may prevent potential damage due to generation of excessive inflammatory immune responses [9, 30]. The down regulation of TLRs, C-C and C-X-C chemokine receptors in lymphocytes of vitD group likely reflects restriction of inflammatory responses. VitD_3_ supplementation of pregnant women at risk of preeclampsia led to a decrease in TLR4 expression and a subsequent decrease in pro-inflammatory cytokine secretion *ex vivo* [31]. Several in vitro studies have shown that 1,25(OH)_2_D_3_ can induce hypo-responsiveness to pathogen-associated molecular patterns (PAMPs) by downregulating expression of TLR2 and TLR4 on monocytes [32, 33]. Human corneal epithelial cells when treated with vitD_3_ (cholecalciferol) led to decreased expression of TLR3 and pro-inflammatory cytokines [34]. Again, intake of active vitD_3_ by asthma patients led to an increased expression of TLR9 but not other TLRs by IL-10 secreting CD4^+^ T cells. The study further showed that, in vitro addition of 1,25(OH)_2_D_3_ could induce expression of TLR9 on IL-10 secreting Treg cells from healthy volunteers [35]. Expression of SOCS5 that negatively regulates cytokines was also down regulated in the vitD group. Cytokine signaling is contained by multiple tiers of control where specific responses elicited by cytokine stimulation, their threshold and magnitude are regulated by numerous mechanisms [36].

HDAC9 has distinct effects on Foxp3 expression and function. Inhibiting HDACs individually or in combination may enhance Treg stability and suppressive function [37, 38]. In the current study, we found down-regulation of HDACs genes in lymphocytes in vitD group that may have similar roles in promoting Treg functions. Down-regulation of genes in the TCR complex, T cell co-stimulatory molecules and major histocompatibility complexes (T cell adaptive immunity) in the vitD group suggests suppression of T cell signaling pathway by vitamin D. Induction of CD2, CD40LG and IL-12RB2 expression that are important in T and NK cell function and inflammatory responses, and down-regulation of receptors for these ligands suggest balanced responses to in vivo vitD_3_ supplementation that would be expected to mitigate major downstream effects. Similarly, induction of IFN-γ expression was paralleled with down-regulation of its receptors.

The study had several limitations. A high percentage (69 %) of the study participants had caesarean delivery and this may affect the generalizability of the study findings even though data were adjusted with mode of delivery. It is important to mention here that rates of caesarean births in Bangladesh have increased from 2 % in 2000 to 17 % in 2011 [39]. According to a recent large population based cross-sectional study (*n* = 21,560) in Bangladesh, 73 % of deliveries conducted in private or charitable health facilities were performed by C-section, more frequently without medical indications [40]. The major reasons were- private providers were motivated by financial incentives to conduct C-sections more often than absolutely necessary; women of higher socioeconomic status were more likely to go for elective caesareans [41], low cost of C-section procedure made it affordable by majority of population. Caesarian and facility based deliveries are heavily subsidized by the government because emergency obstetric care plays an important role in reducing maternal mortality rate in Bangladesh [42]. The non-adherent lymphocytes were a mixture of both T and B lymphocytes but only T cell stimulants were used. Stimulation of CBMC with B cell stimulant or TLR ligands such as LPS might have yielded a more comprehensive picture of vitamin D related cytokine functions. The low sample size for the PCR array analyses may have limited our statistical power to detect significant differences between the vitD and placebo groups. In most of in vitro and *ex vivo* studies and cell models, active form of vitD_3_ has been shown to decrease TLR expression and consequently inflammatory responses [30, 31, 33, 35]. We have not determined the active form of vitD_3_ since the level of this hormone is tightly regulated, has short half-life and does not change with nutritional vitamin D status of the body [43]. It is likely that the in vivo effects of vitD_3_ supplementation have been mediated by intracrine conversion of circulating 25(OH)D_3_ to active form of vitD_3_ [44]. It has been reported that the anti-inflammatory benefits of vitamin D and optimal immune function was seen in individuals with 25(OH)D_3_ as high as 100 nmol/L [30, 45]. In the vitD group, 45 % of the neonates had >100 nmol/L of 25(OH)D_3_ levels which was accompanied by modulation of immune responses evident in the study.

## Conclusion

Antenatal third-trimester supplementation with 35,000 IU/week of vitD_3_ had limited effects on Th1, Th2, Th17 and inflammatory pathways in cord blood. In contrast to in vitro models, the present observations generated from *ex vivo* lymphocytes in the context of a randomized controlled trial do not support the hypothesis that high-dose prenatal vitD_3_ supplementation favors fetal-neonatal Th2 dominance over Th1 responses. Rather, possible modulatory effects of prenatal vitD_3_ on the cord blood cytokine expression appeared to be balanced.

## Abbreviations

25(OH)D_3_, 25-hydroxy-vitamin D_3_; CBA, Cytometric Bead Array; CBMC, cord blood mononuclear cells; CCRs, C-C chemokine receptors; HDAC, histone deacetylases; iCD3/iCD28, anti-CD3/anti-CD28; PHA, phytohemagglutinin; TCR, T cell receptor complex; Th, T helper cell type; TLRs, toll-like receptors; TOLLIP, toll interacting protein; Treg, T regulatory cells; vitD_3_, vitamin D_3_

## Additional file

Additional file 1: Table S1. Demographic characteristics of the study participants. **Table S2.** Reasons for C-section deliveries of the participants included in the present and original cohort. **Table S3.** Description of the functions of some of the genes in the Placebo group as analyzed by PCR Array method. (DOCX 20 kb)

## References

[1] Anderson LN, Chen Y, Omand JA, Birken CS, Parkin PC, To T, Maguire JL, Collaboration TAK: Vitamin D exposure during pregnancy, but not early childhood, is associated with risk of childhood wheezing. J Dev Orig Health Dis. 2015;6(4):308–16. 10.1017/S2040174415001063 25885931

[2] Christesen HT, Elvander C, Lamont RF, Jorgensen JS (2012). The impact of vitamin D in pregnancy on extraskeletal health in children: a systematic review. Acta Obstet Gynecol Scand.

[3] Devereux G, Litonjua AA, Turner SW, Craig LC, McNeill G, Martindale S, Helms PJ, Seaton A, Weiss ST (2007). Maternal vitamin D intake during pregnancy and early childhood wheezing. Am J Clin Nutr.

[4] Maslova E, Hansen S, Jensen CB, Thorne-Lyman AL, Strom M, Olsen SF (2013). Vitamin D intake in mid-pregnancy and child allergic disease - a prospective study in 44,825 Danish mother-child pairs. BMC Pregnancy Childbirth.

[5] Miyake Y, Sasaki S, Tanaka K, Hirota Y (2010). Dairy food, calcium and vitamin D intake in pregnancy, and wheeze and eczema in infants. Eur Respir J.

[6] Pike KC, Inskip HM, Robinson S, Lucas JS, Cooper C, Harvey NC, Godfrey KM, Roberts G, Southampton Women’s Survey Study G (2012). Maternal late-pregnancy serum 25-hydroxyvitamin D in relation to childhood wheeze and atopic outcomes. Thorax.

[7] Wills AK, Shaheen SO, Granell R, Henderson AJ, Fraser WD, Lawlor DA (2013). Maternal 25-hydroxyvitamin D and its association with childhood atopic outcomes and lung function. Clin Exp Allergy.

[8] Gale CR, Robinson SM, Harvey NC, Javaid MK, Jiang B, Martyn CN, Godfrey KM, Cooper C, Princess Anne Hospital Study G (2008). Maternal vitamin D status during pregnancy and child outcomes. Eur J Clin Nutr.

[9] Adams JS, Hewison M (2008). Unexpected actions of vitamin D: new perspectives on the regulation of innate and adaptive immunity. Nat Clin Pract Endocrinol Metab.

[10] Alijotas-Reig J, Llurba E, Gris JM (2014). Potentiating maternal immune tolerance in pregnancy: a new challenging role for regulatory T cells. Placenta.

[11] Warning JC, McCracken SA, Morris JM (2011). A balancing act: mechanisms by which the fetus avoids rejection by the maternal immune system. Reproduction.

[12] Tang J, Zhou R, Luger D, Zhu W, Silver PB, Grajewski RS, Su SB, Chan CC, Adorini L, Caspi RR (2009). Calcitriol suppresses antiretinal autoimmunity through inhibitory effects on the Th17 effector response. J Immunol.

[13] Joshi S, Pantalena LC, Liu XK, Gaffen SL, Liu H, Rohowsky-Kochan C, Ichiyama K, Yoshimura A, Steinman L, Christakos S (2011). 1,25-dihydroxyvitamin D(3) ameliorates Th17 autoimmunity via transcriptional modulation of interleukin-17A. Mol Cell Biol.

[14] Lemire JM, Archer DC, Beck L, Spiegelberg HL (1995). Immunosuppressive actions of 1,25-dihydroxyvitamin D3: preferential inhibition of Th1 functions. J Nutr.

[15] Boonstra A, Barrat FJ, Crain C, Heath VL, Savelkoul HF, O’Garra A (2001). 1alpha,25-Dihydroxyvitamin d3 has a direct effect on naive CD4(+) T cells to enhance the development of Th2 cells. J Immunol.

[16] Vijayendra Chary A, Hemalatha R, Seshacharyulu M, Vasudeva Murali M, Jayaprakash D, Dinesh Kumar B (2015). Vitamin D deficiency in pregnant women impairs regulatory T cell function. J Steroid Biochem Mol Biol.

[17] Barrat FJ, Cua DJ, Boonstra A, Richards DF, Crain C, Savelkoul HF, de Waal-Malefyt R, Coffman RL, Hawrylowicz CM, O’Garra A (2002). In vitro generation of interleukin 10-producing regulatory CD4(+) T cells is induced by immunosuppressive drugs and inhibited by T helper type 1 (Th1)- and Th2-inducing cytokines. J Exp Med.

[18] Roth DE, Al Mahmud A, Raqib R, Akhtar E, Perumal N, Pezzack B, Baqui AH (2013). Randomized placebo-controlled trial of high-dose prenatal third-trimester vitamin D3 supplementation in Bangladesh: the AViDD trial. Nutr J.

[19] Roth DE, Al Mahmud A, Raqib R, Akhtar E, Black RE, Baqui AH (2013). Pharmacokinetics of high-dose weekly oral vitamin D3 supplementation during the third trimester of pregnancy in Dhaka, Bangladesh. Nutrients.

[20] Raqib R, Ly A, Akhtar E, Mily A, Perumal N, Al-Mahmud A, Rekha RS, Hel Baqui A, Roth DE (2014). Prenatal vitamin D(3) supplementation suppresses LL-37 peptide expression in ex vivo activated neonatal macrophages but not their killing capacity. Br J Nutr.

[21] Barrera D, Noyola-Martinez N, Avila E, Halhali A, Larrea F, Diaz L (2012). Calcitriol inhibits interleukin-10 expression in cultured human trophoblasts under normal and inflammatory conditions. Cytokine.

[22] Das M, Tomar N, Sreenivas V, Gupta N, Goswami R (2014). Effect of vitamin D supplementation on cathelicidin, IFN-gamma, IL-4 and Th1/Th2 transcription factors in young healthy females. Eur J Clin Nutr.

[23] Chen S, Sims GP, Chen XX, Gu YY, Chen S, Lipsky PE (2007). Modulatory effects of 1,25-dihydroxyvitamin D3 on human B cell differentiation. J Immunol.

[24] Lemire JM, Adams JS, Sakai R, Jordan SC (1984). 1 alpha,25-dihydroxyvitamin D3 suppresses proliferation and immunoglobulin production by normal human peripheral blood mononuclear cells. J Clin Invest.

[25] Chambers ES, Suwannasaen D, Mann EH, Urry Z, Richards DF, Lertmemongkolchai G, Hawrylowicz CM (2014). 1alpha,25-dihydroxyvitamin D3 in combination with transforming growth factor-beta increases the frequency of Foxp3(+) regulatory T cells through preferential expansion and usage of interleukin-2. Immunology.

[26] Chambers ES, Nanzer AM, Richards DF, Ryanna K, Freeman AT, Timms PM, Martineau AR, Griffiths CJ, Corrigan CJ, Hawrylowicz CM (2012). Serum 25-dihydroxyvitamin D levels correlate with CD4(+)Foxp3(+) T-cell numbers in moderate/severe asthma. J Allergy Clin Immunol.

[27] Smolders J, Menheere P, Thewissen M, Peelen E, Tervaert JW, Hupperts R, Damoiseaux J (2010). Regulatory T cell function correlates with serum 25-hydroxyvitamin D, but not with 1,25-dihydroxyvitamin D, parathyroid hormone and calcium levels in patients with relapsing remitting multiple sclerosis. J Steroid Biochem Mol Biol.

[28] Urry Z, Chambers ES, Xystrakis E, Dimeloe S, Richards DF, Gabrysova L, Christensen J, Gupta A, Saglani S, Bush A (2012). The role of 1alpha,25-dihydroxyvitamin D3 and cytokines in the promotion of distinct Foxp3+ and IL-10+ CD4+ T cells. Eur J Immunol.

[29] Klug-Micu GM, Stenger S, Sommer A, Liu PT, Krutzik SR, Modlin RL, Fabri M (2013). CD40 ligand and interferon-gamma induce an antimicrobial response against Mycobacterium tuberculosis in human monocytes. Immunology.

[30] Calton EK, Keane KN, Newsholme P, Soares MJ (2015). The impact of Vitamin D levels on inflammatory status: a systematic review of immune cell studies. PLoS One.

[31] Qian L, Wang H, Wu F, Li M, Chen W, Lv L (2015). Vitamin D3 alters Toll-like receptor 4 signaling in monocytes of pregnant women at risk for preeclampsia. Int J Clin Exp Med.

[32] Di Rosa M, Malaguarnera G, De Gregorio C, Palumbo M, Nunnari G, Malaguarnera L (2012). Immuno-modulatory effects of vitamin D3 in human monocyte and macrophages. Cell Immunol.

[33] Sadeghi K, Wessner B, Laggner U, Ploder M, Tamandl D, Friedl J, Zugel U, Steinmeyer A, Pollak A, Roth E (2006). Vitamin D3 down-regulates monocyte TLR expression and triggers hyporesponsiveness to pathogen-associated molecular patterns. Eur J Immunol.

[34] Reins RY, Baidouri H, McDermott AM (2015). Vitamin D activation and function in human corneal epithelial cells during tlr-induced inflammation. Invest Ophthalmol Vis Sci.

[35] Urry Z, Xystrakis E, Richards DF, McDonald J, Sattar Z, Cousins DJ, Corrigan CJ, Hickman E, Brown Z, Hawrylowicz CM (2009). Ligation of TLR9 induced on human IL-10-secreting Tregs by 1alpha,25-dihydroxyvitamin D3 abrogates regulatory function. J Clin Invest.

[36] Croker BA, Kiu H, Nicholson SE (2008). SOCS regulation of the JAK/STAT signalling pathway. Semin Cell Dev Biol.

[37] Pan F, Fan H, Liu Z, Jiang S (2012). T cell signaling targets for enhancing regulatory or effector function. Sci Signal.

[38] Guo X, Jie Y, Ren D, Zeng H, Zhang Y, He Y, Pan Z (2012). Histone deacetylase inhibitors promote mice corneal allograft survival through alteration of CD4+ effector T cells and induction of Foxp3+ regulatory T cells. Cell Immunol.

[39] BDHS (2013). Bangladesh demographic and health survey 2011: National Institute of Population Research and Training.

[40] Neuman M, Alcock G, Azad K, Kuddus A, Osrin D, More NS, Nair N, Tripathy P, Sikorski C, Saville N (2014). Prevalence and determinants of caesarean section in private and public health facilities in underserved South Asian communities: cross-sectional analysis of data from Bangladesh, India and Nepal. BMJ Open.

[41] Saha L, Chowdhury SB (2011). Study on primary cesarean section. Mymensingh Med J.

[42] Koblinsky M, Anwar I, Mridha MK, Chowdhury ME, Botlero R (2008). Reducing maternal mortality and improving maternal health: Bangladesh and MDG 5. J Health Popul Nutr.

[43] Zerwekh JE (2008). Blood biomarkers of vitamin D status. Am J Clin Nutr.

[44] Hewison M (2011). Antibacterial effects of vitamin D. Nat Rev Endocrinol.

[45] Ojaimi S, Skinner NA, Strauss BJ, Sundararajan V, Woolley I, Visvanathan K (2013). Vitamin D deficiency impacts on expression of toll-like receptor-2 and cytokine profile: a pilot study. J Transl Med.

