# Supplementary material for: Prenatal high-dose vitamin D3 supplementation has balanced effects on cord blood Th1 and Th2 responses
Source: Nutr J. 2016 Aug 9;15:75. doi: 10.1186/s12937-016-0194-5 (PMC4979151; doi:10.1186/s12937-016-0194-5)
Supplement: Additional file 1: Table S1. — Demographic characteristics of the study participants. Table S2. Reasons for C-section deliveries of the participants included in the present and original cohort. Table S3. Description of the functions of some of the genes in the Placebo group as analyzed by PCR Array method. (DOCX 20 kb) [file 12937_2016_194_MOESM1_ESM.docx]

**Prenatal high-dose vitamin D_3_ supplementation has balanced effects on cord blood Th1 and Th2 responses**

Evana Akhtar^1^, Akhirunnesa Mily^1^, Md. Ahsanul Haq^1^, Abdullah Al-Mahmud^2^, Shams El-Arifeen^2^, Abdullah Hel-Baqui^3^, Daniel E. Roth^4^, Rubhana Raqib^1^*

^1^Immunobiology, Nutrition and Toxicology Laboratory, Infectious Diseases Division, International Centre for Diarrhoeal Disease Research, Bangladesh, 1212 Dhaka, Bangladesh

^2^Maternal and Child Health Division, International Centre for Diarrhoeal Disease Research, Bangladesh, 1212 Dhaka, Bangladesh

^3^International Center for Maternal and Newborn Health, Department of International Health, The Johns Hopkins Bloomberg School of Public Health, 615 North Wolfe Street, Baltimore, MD 21205, USA

^4^Department of Pediatrics, Hospital for Sick Children and University of Toronto, 555 University Avenue, Toronto, ON M5G 1X8, Canada.

**Short title:** Prenatal vitamin D_3_ and neonatal immunity

***Corresponding author:** Rubhana Raqib, Immunobiology, Nutrition and Toxicology Laboratory, Infectious Diseases Division, International Centre for Diarrhoeal Disease Research, Bangladesh (icddr,b), Mohakhali, Dhaka-1212, Bangladesh. Tel.: +880-2-9827068; Fax: +880-28812529.

**E-mail address:** [rubhana@icddrb.org](mailto:rubhana@icddrb.org)

**Trial Registration:**

ClinicalTrials.gov (NCT01126528).

**Table S1** Demographic characteristics of the study participants.

| Variables | Original cohort  (n=160) | Study cohort  (n=80) | PCR Array cohort (n=12) |
| --- | --- | --- | --- |
| Variables | Mean ± SD | Mean ± SD | Mean ± SD |
| Maternal Age, years | 22.43±3.51 | 22.64±2.61 | 20.92±2.70 |
| Gestational age at birth, week | 38.37±2.53 | 39.27±2.48 | 40.08±2.24 |
| Delivery Mode n (%) |  |  |  |
| Vaginal | 59 (40.0%) | 25 (31.3%) | 1 (0.0%) |
| C-section | 88 (60.0%) | 28 (68.8%) | 11 (100.0%) |
| Birth weight, gm | 2941.88±1121.53 | 2865.37±444.94 | 2880.67±478.71 |
| Male : female | 70:77 | 34:56 | 5:7 |
| Level of education, n (%) |  |  |  |
| No education | 15 (9.4%) | 8 (10.0%) | 1 (8.3%) |
| Primary school incomplete (<8 yrs) | 88 (55.0%) | 44 (55.0%) | 9 (75.0%) |
| High school incomplete (> = 8 to <12 yrs) | 48 (30.0%) | 25 (28.7%) | 2 (16.7%) |
| Graduate school (> = 12 yrs) | 9 (5.6%) | 4 (6.3%) | 0 |
| Primary Occupation, n (%) |  |  |  |
| Homemaker | 141 (88.1%) | 69 (86.3%) | 11 (91.7%) |
| Others | 19 (11.9%) | 11 (13.7%) | 1 (8.3%) |
| Floor material of house, n (%) |  |  |  |
| Earth or bamboo | 10 (6.3%) | 2 (2.5%) | 0 |
| Wood | 4 (2.5%) | 1 (1.3%) | 0 |
| Cement or concrete | 146 (91.3%) | 77 (96.3%) | 12 (100.0%) |
| Wall material of house, n (%) |  |  |  |
| Earth or bamboo | 6 (3.8%) | 5 (6.3%) | 1 (8.3%) |
| Tin | 38 (23.8%) | 15 (18.8%) | 2 (16.7%) |
| Cement or concrete | 116 (72.5%) | 60 (75.0%) | 9 (75.0%) |

Note: Data expressed as means ± standard deviations and or number with percentage in parentheses.

^a^Student’s t test was used for calculating p values for maternal Age, Gestational age at birth and birth weight.

^b^P values were calculated for delivery mode between two supplementation using χ^2^test.

^c^Fisher Exact test was applied for comparisons between the two group.

**Table S2** Reasons for C-section deliveries of the participants included in the present and original cohort [[1](#_ENREF_1)]

| Reason for C-section | Study Cohort (n=55) | Original cohort [[1](#_ENREF_1)] (n=88) |
| --- | --- | --- |
| ^1^Fetal distress | 30 (54.55%) | 48 (54.55%) |
| Oligohydramnios | 8 (14.55%) | 11 (12.50%) |
| Post term pregnancy | 2 (3.64%) | 2 (2.27%) |
| Elective | 3 (5.45%) | 5 (5.68%) |
| Previous history of C-section | 6 (10.91%) | 11 (12.50%) |
| ^2^Others | 4 (7.27%) | 7 (7.95%) |
| ^3^Missing | 2 (3.64%) | 4 (4.55%) |

^1^Including breech / transverse fetal presentation and reduced fetal movement.

^2^Includes increased fetal movement (n=2); maternal hypertension (n=4); cephalopelvic disproportion (CPD)(n=1), congenital femoral deficiency (CFD) (n=1)

^3^Reasons for performing C-section are not available.

**Table S3** Description of the functions of some of the genes in the Placebo group as analyzed by PCR Array method

| Gene  Symbol | Description | Fold up- or  down-regulation | P-value |
| --- | --- | --- | --- |
| CD2 | CD2 molecule | -1.28 | 0.34 |
| CD3D | CD3d molecule, delta (CD3-TCR complex) | -2.55 | 0.33 |
| CD3G | CD3g molecule, gamma (CD3-TCR complex) | -3.42 | 0.33 |
| CD40LG | CD40 ligand | -1.43 | 0.34 |
| CD81 | CD81 molecule | -5.24 | 0.33 |
| CD8B | CD8b molecule | -3.92 | 0.34 |
| CLEC7A | C-type lectin domain family 7, member A | -45.49 | 0.34 |
| CXCR4 | Chemokine (C-X-C motif) receptor 4 | -5.70 | 0.31 |
| HDAC4 | Histone deacetylase 4 | -7.62 | 0.34 |
| HLADRA | Major histocompatibility complex, class II, DR alpha | -9.23 | 0.21 |
| IFNG | Interferon, gamma | 1.10 | 0.35 |
| IFNGR1 | Interferon gamma receptor 1 | -6.81 | 0.33 |
| IFNGR2 | Interferon gamma receptor 2 (interferon gamma transducer 1) | -8.11 | 0.34 |
| IGBP1 | Immunoglobulin (CD79A) binding protein 1 | -4.10 | 0.34 |
| IL12RB2 | IL-12 receptor, beta 2 | -1.86 | 0.34 |
| IL2RA | Interleukin 2 receptor, alpha | 1.68 | 0.35 |
| IRF4 | Interferon regulatory factor 4 | 1.01 | 0.35 |
| KLF6 | Kruppel-like factor 6 | -5.30 | 0.33 |
| NCK1 | NCK adaptor protein 1 | -6.90 | 0.34 |
| NCK2 | NCK adaptor protein 2 | -4.32 | 0.34 |
| TGFB1 | Transforming growth factor, beta 1 | -3.66 | 0.33 |

Data has not been provided for some genes when a particular gene’s relative expression level is low in both control and stimulated groups (>30 threshold cycles); gene expression level for a series of genes in the vitD group was reasonably high, but very low in Placebo group; hence the placebo data for those genes were not included in Table S3.

1. Roth DE, Al Mahmud A, Raqib R, Akhtar E, Perumal N, Pezzack B, Baqui AH: **Randomized placebo-controlled trial of high-dose prenatal third-trimester vitamin D3 supplementation in Bangladesh: the AViDD trial**. *Nutr J* 2013, **12**(1):47.
